# Evolution of reciprocity with limited payoff memory

**DOI:** 10.1098/rspb.2023.2493

**Published:** 2024-06-19

**Authors:** Nikoleta E. Glynatsi, Alex McAvoy, Christian Hilbe

**Affiliations:** ^1^ Max Planck Research Group on the Dynamics of Social Behavior, Max Planck Institute for Evolutionary Biology, 24306 Plön, Germany; ^2^ School of Data Science and Society, University of North Carolina at Chapel Hill, Chapel Hill, NC 27599, USA; ^3^ Department of Mathematics, University of North Carolina at Chapel Hill, Chapel Hill, NC 27599, USA

**Keywords:** direct reciprocity, evolution of cooperation, evolutionary dynamics, repeated Prisoner’s Dilemma, social learning

## Abstract

Direct reciprocity is a mechanism for the evolution of cooperation in repeated social interactions. According to the literature, individuals naturally learn to adopt conditionally cooperative strategies if they have multiple encounters with their partner. Corresponding models have greatly facilitated our understanding of cooperation, yet they often make strong assumptions on how individuals remember and process payoff information. For example, when strategies are updated through social learning, it is commonly assumed that individuals compare their average payoffs. This would require them to compute (or remember) their payoffs against everyone else in the population. To understand how more realistic constraints influence direct reciprocity, we consider the evolution of conditional behaviours when individuals learn based on more recent experiences. Even in the most extreme case that they only take into account their very last interaction, we find that cooperation can still evolve. However, such individuals adopt less generous strategies, and they cooperate less often than in the classical setup with average payoffs. Interestingly, once individuals remember the payoffs of two or three recent interactions, cooperation rates quickly approach the classical limit. These findings contribute to a literature that explores which kind of cognitive capabilities are required for reciprocal cooperation. While our results suggest that some rudimentary form of payoff memory is necessary, it suffices to remember a few interactions.

## 1. Introduction

Evolutionary game theory describes the dynamics of populations when an individual’s fitness depends on the traits or strategies of other population members [1–4]. This theory can be used to describe the dynamics of animal conflict [5], of cancer cells [6] and of cooperation [7]. Respective models translate strategic interactions into games [8]. These games specify how individuals (players) interact, which strategies individuals can choose, and what fitness consequences (or payoffs) the different strategies have. In addition, these models also specify the mode by which successful strategies spread over time. In models of biological evolution, individuals with a high fitness produce more offspring; in models of cultural evolution, such individuals are imitated more often. Although biological and cultural evolution are sometimes treated as equivalent, there can be important differences [9–11]. For example, models of biological evolution do not require individuals to have any particular cognitive abilities. Here, it is the evolutionary process itself that biases the population towards strategies with higher fitness. By contrast, in models of cultural evolution, individuals need to be aware of the different strategies present in the population, and they need to identify those strategies with a higher payoff. As a consequence, evolutionary outcomes may depend on how easily different behaviours can be learned [12], as well as on how readily payoffs can be compared.

These difficulties to learn strategies by social imitation are particularly pronounced in models of direct reciprocity. This literature follows Trivers’ insight that individuals have more of an incentive to cooperate in social dilemmas when they interact repeatedly [13]. In repeated interactions, individuals can condition their behaviour on their past experiences with their interaction partner. They may use strategies such as Tit-for-Tat [14,15] or Generous Tit-for-Tat [16,17] to preferentially cooperate with other cooperators. Such conditional strategies approximate human behaviour fairly well [18–22] and they have also been documented in several other species [23–25]—although direct reciprocity is generally more difficult to demonstrate in animals [26–28]. However, at the outset, it is not clear how easy it is to *learn* reciprocal strategies by social imitation. As one obstacle, even if others’ strategies are perfectly observable, individuals might find it difficult to identify which ones have the highest payoff. After all, the payoff of a strategy of direct reciprocity is not determined by the outcome of any single round. Rather, it is determined by how well this strategy fares over an entire sequence of rounds, against many different population members. In practice, such information might be difficult both to obtain and to process.

Most models of direct reciprocity do not address these difficulties [29–46] and, instead, assume individuals can easily copy the strategies of others. Similarly, they assume that updating decisions are based on the strategies’ average (or expected) payoffs, which are based on all rounds and all interactions. These assumptions create a curious inconsistency in how models represent an individual’s cognitive abilities. On the one hand, when playing the game, individuals are often assumed to have restricted memory. Respective studies typically assume that individuals make their decisions each round based on the outcome of the last round only (with only a few exceptions, see [47–52]). Yet when learning new strategies, individuals are assumed to remember (or compute) each others’ precise average payoff across many rounds and many interaction partners. Herein, we wish to explore whether this latter assumption is actually necessary for the evolution of reciprocity through social imitation. We ask whether individuals can learn to adopt reciprocal strategies even when learning is based on payoff information from a limited number of rounds.

To explore that question, we theoretically study imitation dynamics in the repeated Prisoner’s Dilemma, using two extreme scenarios. The first scenario is the classical modelling approach. Here, individuals update their strategies based on their expected payoffs. We contrast this model with an alternative scenario where individuals update their strategies based on the very last (one-shot) payoff they obtained. We find that individuals with limited payoff memory tend to adopt less generous strategies. Yet moderate levels of cooperation can still evolve. Moreover, as we increase the individuals’ payoff memory to include the last two or three one-shot payoffs, cooperation rates quickly approach the rates observed in the classical baseline.

## 2. Model and methods

To explore the impact of limited payoff memory, we adapt existing models of the evolution of direct reciprocity. These models involve two different time scales. The short time scale describes the game dynamics. Here, individuals with fixed strategies are randomly matched to interact with each other in repeated social dilemmas. The long time scale describes the evolutionary dynamics. Here, individuals can update their repeated-game strategies based on the payoffs they yield. In the following, we introduce the basic setup of our model; all details and derivations are described in the electronic supplementary material.

### (a) Description of the game dynamics

We consider a well-mixed population consisting of *N* players. Players are randomly matched in pairs to participate in a repeated donation game [53] with their respective co-player. Each round, they can either cooperate (C) or defect (D). A cooperating player provides a benefit *b* to the other player at their own cost *c*, with 0 < *c* < *b*. A defecting player provides no benefit and pays no cost. Thus, the players’ payoffs in a single round are given by the matrix2.1

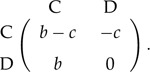
In particular, payoffs take the form of a Prisoner’s Dilemma: mutual cooperation yields a better payoff than mutual defection (*b* − *c* > 0), but each player individually prefers to defect, independent of the co-player’s action (*b* > *b* − *c* and 0 > −*c*). To incorporate repetition, we assume that after each round, there is a constant continuation probability *δ* of interacting for another round. For *δ* = 0, we recover the case of a conventional (one-shot) Prisoner’s Dilemma. Here, mutual defection is the only equilibrium. As *δ* increases, the game turns into a repeated game. Here, additional equilibria emerge, with some of them allowing for full cooperation [54–57].

In a one-shot donation game, players can only choose among two pure strategies (they can either cooperate or defect). In the repeated game, strategies become arbitrarily complex. Here, strategies are contingent rules, telling players what to do depending on the outcome of all previous rounds. For simplicity, in the following, we assume individuals use *reactive strategies* [17]. A reactive strategy only depends on the other player’s action in the last round. Such strategies can be written as a three-dimensional tuple *s* = (*y*, *p*, *q*). The first entry *y* is the probability that the player opens with cooperation in the first round. The two other entries are the probabilities that the player cooperates in all subsequent rounds, depending on whether the co-player cooperated (*p*) or defected (*q*) in the previous round. The set of reactive strategies is simple enough to facilitate an explicit mathematical analysis [1]. Yet it is rich enough to capture several important strategies of repeated games. For example, it contains ALLD =(0,0,0), the strategy that always defects. Similarly, it contains Tit-for-Tat, TFT =(1,1,0), the strategy that copies the co-player’s previous action (and that cooperates in the first round). Finally, it contains Generous Tit-for-Tat, GTFT=(1,1,q), where *q* > 0 reflects a player’s generosity in response to a co-player’s defection [16,17].

In the short run, the players’ strategies are taken to be fixed. Players use their strategies to decide whether to cooperate in a series of repeated games against all other population members. In the long run, however, the players’ strategies may change depending on the payoffs they yield, as we describe in the following.

### (b) Description of the evolutionary dynamics

Herein, we assume population members update their strategies based on social learning. To model these strategy updates, we consider a pairwise comparison process [58]. This process assumes that at regular time intervals, one population member is randomly selected, and given the chance to revise its strategy. We refer to this player as the ‘learner’. With probability *μ* (reflecting a mutation rate), the learner simply adopts a random strategy (all reactive strategies have the same probability to be chosen). With the converse probability 1 − *μ*, the learner randomly picks a ‘role model’ from the population. The learner then compares its own payoff *π*_L_ from the repeated game to the role model’s payoff *π*_RM_. The learner adopts the role model’s strategy with a probability *φ* described by a Fermi function [59,60],

2.2 $$\varphi (\pi_{L},\pi_{\mathrm{RM}})=\frac{1}{1+e^{-\beta (\pi_{\mathrm{RM}}-\pi_{L})}}.$$

The selection strength parameter *β* ≥ 0 indicates how sensitive players are to payoff differences. For *β* = 0, payoff differences are irrelevant, and the learner simply adopts the role model’s strategy with probability 1/2. As the selection strength *β* increases, players are increasingly biased to imitate the role model only if it has the higher payoff.

**Figure 1.**
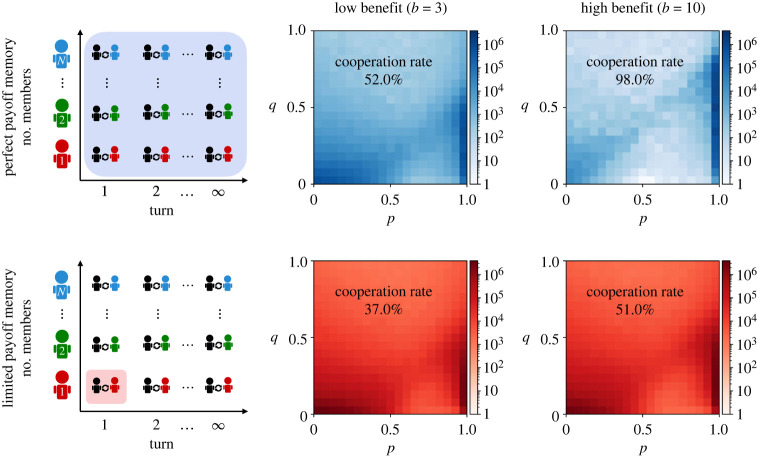
Evolutionary dynamics under perfect and limited payoff memory. The leftmost panels give a schematic overview of the two main scenarios we compare. The two scenarios differ in how many past interactions individuals take into account when updating their strategy. In the scenario with perfect payoff memory, individuals consider all their past interactions (against all population members, and taking every turn of each repeated game into account). In the scenario with limited payoff memory, individuals only consider their very last interaction (against one specific population member, taking into account only one round of the repeated game). The four panels on the right side depict the outcome of evolutionary simulations for repeated games with either a low or a high benefit of cooperation. Colours represent how often the respective region of the strategy space is visited over time. In all four panels, two regions are visited particularly often. One region corresponds to a neighbourhood of ALLD with *p* ≈ *q* ≈ 0 (lower left corner). The other region corresponds to a strip of conditionally cooperative strategies with *p* ≈ 1 and *q* satisfying the constraints (3.1) and (3.2), respectively (lower right corner). The resulting average cooperation rate depends on which of these two neighbourhoods is visited more often. Simulations are run for *T* = 10^7^ time steps, using a cost *c* = 1, a continuation probability of *δ* = 0.999 and a selection strength of *β* = 1, in a population of size *N* = 100.

We deviate from previous models in how we interpret the payoffs *π*_L_ and *π*_RM_, which form the basis of the pairwise comparisons in equation (2.2). In previous work, these payoffs are taken to be the respective players’ expected payoffs. We interpret that setup as a model with perfect payoff memory. There, the payoffs *π*_L_ and *π*_RM_ represent an average over all possible repeated games the two individuals have played with all population members (figure 1, upper left panel). The use of expected payoffs is mathematically convenient, because explicit formulae for these payoffs are available [1]. Herein, we compare this model of perfect payoff memory with a model with limited payoff memory. In the latter model, the players’ payoffs *π*_L_ and *π*_RM_ are taken to be the payoffs that each player received in their very last round prior to making social comparisons. That is, players only consider the very last repeated game they participated in, and there they only take into account the outcome of the very last round (figure 1, lower left panel). This assumption could reflect, for example, a strong recency bias in how individuals evaluate payoffs. In addition to this extreme case of limited payoff memory, later on we also explore cases in which players take into account the outcome of two, three, four or more recent rounds.

In both the cases of perfect and limited memory, we iterate the elementary strategy update step described above many times. This gives rise to a stochastic process that describes which strategies players adopt over time. Provided the selection strength is finite, this process satisfies the mathematical property of being ergodic. This implies that in the long run, the time average of the players’ cooperation rates converges, and that this limit is independent of the initial population. We explore the dynamics of this process mathematically and with numerical simulations. These simulations are run sufficiently long for convergence to occur (we numerically checked that independent runs are within a 1% error tolerance). For the results presented in the following, we assume that mutations are rare (*μ* → 0). This assumption is fairly common in evolutionary game theory, both because it makes some computations more efficient [61–63] and because the results can be interpreted more easily. However, in electronic supplementary material, §S3 we show that our main results continue to hold for strictly positive mutation rates.

## 3. Results

### (a) Stability of cooperative populations

To get some intuition for the differences between perfect and limited payoff memory, we first analyse when full cooperation is stable in either scenario. For a population to be fully cooperative, players need to cooperate in the first round (that is, *y* = 1) and after any round with mutual cooperation (that is, *p* = 1). We refer to such a strategy as Generous Tit-for-Tat, GTFT =(1,1,q). To explore whether such a population consisting of GTFT players is stable, we introduce a single mutant who adopts ALLD. We say (*full*) *cooperation is stochastically stable* if the single mutant is more likely to imitate the residents than *vice versa*. For simplicity, we consider a large population (*N* → ∞) and strong selection (*β* → ∞). More general results are derived in the electronic supplementary material.

In the case of perfect payoff memory, it is straightforward to characterize when cooperation is stochastically stable. Here, we simply need to compute the players’ expected payoffs. Because the population mostly consists of residents, and because residents mutually cooperate with each other, their expected payoff is $\pi_{GTFT}=b-c$. On the other hand, the defecting mutant only interacts with residents. Given the residents’ strategy, the mutant receives a benefit *b* in the first round, and in every subsequent round with probability *q*. As a result, the mutant’s expected payoff is $\pi_{ALLD}=(1-\delta +\delta q)b$. For perfect payoff memory, the requirement for cooperation to be stochastically stable reduces to the condition $\pi_{GTFT}>\pi_{ALLD}$, which yields

3.1 $$q<1-\frac{c}{\delta b}.$$

In particular, we recover the previous observation that *q* = 1 − *c*/(*δb*) is the maximum generosity that cooperators should have [16,17,64]. Moreover, because *q* needs to be non-negative, we also conclude that cooperation can only be stable if *δ* > *c*/*b*. Again, this condition for the feasibility of direct reciprocity already exists in the literature [7].

The logic of the case with limited payoff memory is somewhat different. Here, we need to compute how likely it is that each player obtains one of the four possible payoffs {*b* − *c*, −*c*, *b*, 0} in the very last round of a game, before it makes social comparisons. Because residents almost always interact with other residents, their last one-shot payoff is $\pi_{GTFT}=b-c$ almost surely. For the defecting mutant, there are two possibilities. If the mutant’s co-player happens to cooperate in the last round, the mutant receives $\pi_{ALLD}=b$. This case occurs with probability 1 − *δ* + *δq*. On the other hand, if the co-player defects in the last round, the mutant receives $\pi_{ALLD}=0$. This occurs with the converse probability *δ*(1 − *q*). Because *b* − *c* < *b*, residents tend to imitate the mutant in the first case. Because *b* − *c* > 0, mutants tend to imitate the resident in the second case. Cooperation is stochastically stable if the first case is less likely than the second, which yields the condition

3.2 $$q<1-\frac{1}{2\delta }.$$

Interestingly, this condition no longer depends on the exact payoff values, *b* and *c*. This independence arises because of our assumption of strong selection, in which case only the payoff ordering *b* > *c* > 0 matters. Because *q* is non-negative, condition (3.2) can be satisfied only if *δ* > 1/2. That is, players need to interact in more than two rounds, on average.

By comparing the two cases, we find that payoff memory affects whether a conditionally cooperative strategy (1, 1, *q*) is viable. With perfect memory, the maximum generosity *q* must satisfy equation (3.1). In particular, this generosity can become arbitrarily large, provided the game’s benefit-to-cost ratio *b*/*c* and the continuation probability *δ* are sufficiently large. By contrast, with limited payoff memory, the maximum generosity is bounded by 1/2, and it is independent of the benefit-to-cost ratio.

### (b) Evolutionary dynamics of reciprocity

The previous static observations describe whether full cooperation, once established, can be sustained. In a next step, we explore in which cases cooperation actually evolves. To this end, we turn to simulations. We have run separate simulations for perfect and limited payoff memory. In each case, we consider both a low and a high benefit of cooperation (*b*/*c* = 3 and *b*/*c* = 10, respectively). For each simulation, we record which strategies (*y*, *p*, *q*) the players adopt over time. Figure 1 depicts the conditional cooperation probabilities *p* and *q* (we omit the opening move *y* because we use a discount factor *δ* close to 1, such that first-round behaviour is largely irrelevant). In all simulations, we find that the players’ strategies cluster in two regions of the strategy space. The first region corresponds to a neighbourhood of ALLD with (*p*, *q*) ≈ (0, 0). The second region corresponds to a thin strip of cooperative strategies with (*p*, *q*) ≈ (1, *q*). Within this strip, we observe that most strategies satisfy the constraints on *q* suggested by the inequalities (3.1) and (3.2). That is, with perfect memory, most evolving strategies have *q* < 1 − *c*/*b*, whereas with limited payoff memory, most strategies have *q* < 1/2. In particular, for limited payoff memory, changes in the benefit parameter have no effect on the qualitative distribution of strategies.

In each case, the evolutionary dynamics follow a similar cyclic pattern (as described in [17,33]: Resident populations of defectors are most likely invaded by strategies close to TFT. Once the population adopts conditionally cooperative strategies (1, 1, *q*), neutral drift may introduce larger values of generosity *q*. If the resident’s generosity *q* violates the conditions (3.1) and (3.2), defectors can re-invade, and the cycle starts again. The relative time spent near ALLD and near the strip of conditionally cooperative strategies depends on the memory (electronic supplementary material, figure S3, depicting the case of high benefits). For perfect memory, we find that ALLD is replaced relatively quickly by more cooperative strategies. Here, it takes on average 159 invading mutants until ALLD is successfully replaced. By contrast, for limited memory, ALLD is more robust, resisting on average 798 mutant strategies. This picture reverses for an initial population that adopts GTFT. Such populations are much more robust under perfect memory than they are under limited memory. Overall, we find that the impact of memory on the population’s average cooperation rate is substantial. For perfect memory, this rate is 52% for low benefits, and 98% for high benefits. For limited payoff memory, the evolving cooperation rates are smaller but still strictly positive, with 37% cooperation for low benefits and 51% cooperation for high benefits (figure 1).

**Figure 2.**
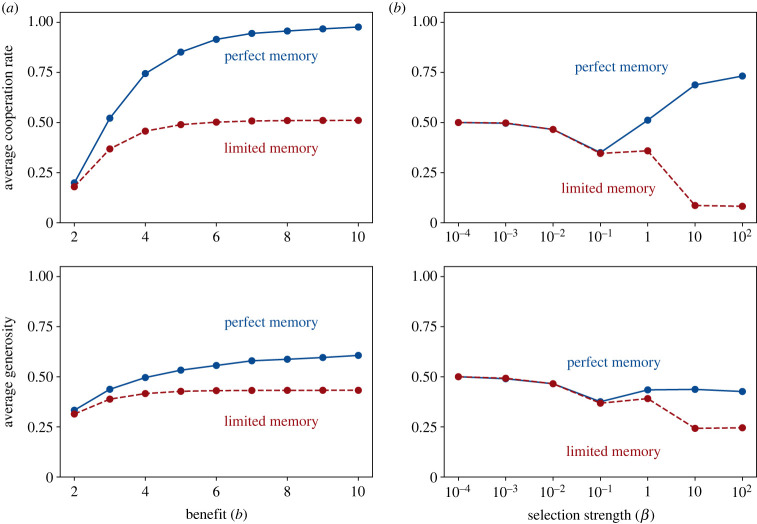
Evolution of direct reciprocity for different parameter values. To explore the robustness of our results, we have run simulations for different benefit values (left panels, *a*), and for different selection strengths (right panels, *b*). In each case, we record the resulting average cooperation rate over the entire simulation (upper panels). In addition, we record the individuals’ average generosity. Here, we only take into account those residents with *p* ≈ 1, and we compute the average of their cooperation probability *q*. These simulations suggest that perfect payoff memory consistently leads to more cooperation and more generosity. Unless explicitly varied, the parameters of the simulation are *N* = 100, *b* = 3, *c* = 1, *β* = 1, *δ* = 0.99. Simulations are run for *T* = 5 × 10^7^ time steps, and each point represents a single simulation run.

To further investigate the influence of different parameters, we have systematically varied the benefit *b* and the selection strength *β* in figure 2. According to figure 2*a*, perfect memory consistently results in a higher cooperation rate, and this relative advantage further increases with an increasing benefit *b*. Interestingly, for limited payoff memory, the cooperation rate remains stable at approximately 50% once *b* ≥ 5. This finding is reminiscent of our earlier static results, which also suggested that changes in the game’s benefits may have a negligible role on the stochastic stability of full cooperation. With respect to the effect of different selection strengths, figure 2*b* suggests that both perfect and limited payoff memory yield similar cooperation rates for weak selection (*β* ≪ 1). This is not a coincidence: it is known that, under weak selection, stochastic payoffs can be replaced by their (deterministic) expectations without altering the evolutionary dynamics [65]—and perfect payoff memory corresponds to the expected value of the payoffs in the limited payoff memory model, owing to the law of large numbers. Beyond weak selection, increasing selection has a positive effect under perfect payoff memory, but a negative effect under limited payoff memory.

### (c) The effect of increasing individual payoff memory

So far, we have taken a rather extreme interpretation of limited payoff memory. We assumed that individuals update their strategies based on their experience in a single round of the Prisoner’s Dilemma, against a single co-player. The limited payoff memory framework can be expanded in various ways. In particular, individuals may recall a larger number of rounds, they may recall their interactions with several co-players, or both. To gain further insights on the impact of payoff memory, we explore four additional scenarios. In the first scenario, players recall the payoffs they obtained in the last two rounds against a single co-player. In the second scenario, players recall their last-round payoffs against two co-players. In the third scenario, they recall the two last rounds against two co-players. Finally, in the last scenario, players update based on the average payoff they receive over all rounds with a single co-player. (Further extensions are possible, but we do not explore them here.)

For most scenarios, we can again derive an analytical condition for when cooperation is stochastically stable. As before, we assume populations are large and that selection is strong. For simplicity, we also assume that the game continues almost certainly after each round (i.e. *δ* approaches 1). The details of this analysis can be found in the electronic supplementary material. In the first two scenarios, we interestingly find that for *b* > 2*c*, cooperation is stochastically stable when $q<\sqrt{2}/2\approx 0.707$. Comparing this condition with the more stringent condition in equation (3.2) suggests that there are now more conditionally cooperative strategies that can sustain cooperation. Hence, cooperation should evolve more easily. In the last scenario, we find that cooperation is stochastically stable when *q* < 1 − (*c*/*b*), which is the same condition as in equation (3.1), even though only a single co-player is considered instead of the whole population.

**Figure 3.**
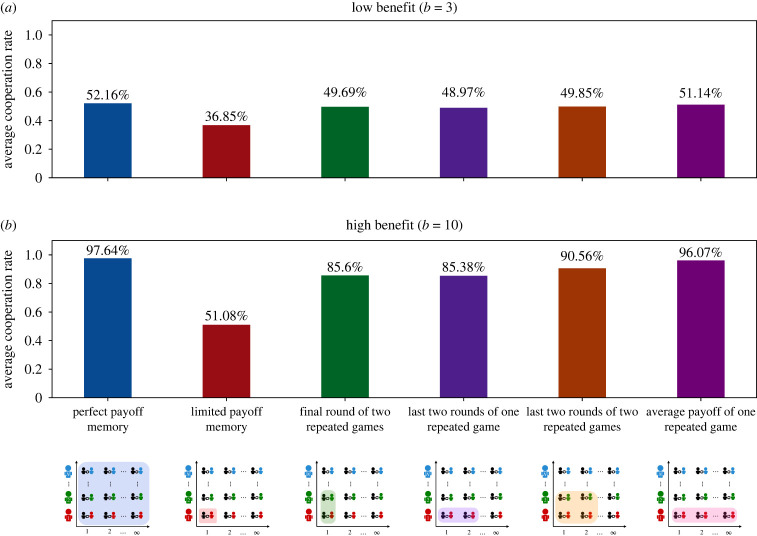
Average cooperation rates for different payoff memories. We vary how much information individuals take into account when updating their strategies. From left to right, we consider the following cases. Updating occurs (i) based on expected payoffs (perfect memory), (ii) based on the last round of one interaction (limited memory), (iii) based on the last round of two interactions, (iv) based on the last two rounds of one interaction, (v) based on the last two rounds of two interactions, and (vi) based on the average payoff of one interaction. Again, simulations are run either for a comparably low benefit of cooperation (*b*/*c* = 3), or for a high benefit (*b*/*c* = 10). We observe that perfect memory always yields the highest cooperation rate. However, when individuals take into account at least two past interactions—cases (iii) to (vi)—evolving cooperation rates are close to this optimum. Simulations are run for *T* = 10^7^ time steps, based on the parameters *N* = 100, *c* = 1, *β* = 1, *δ* = 0.999.

We complement these analytical results with additional simulations; see figure 3. We observe that a minimal increase in the players’ payoff memory (compared with the baseline case with a single round recalled) can promote cooperation considerably. Specifically, in all four scenarios with extended memory, we see similar cooperation rates that approach the rates observed under perfect memory. These results suggest that while it takes *some* payoff memory to sustain substantial cooperation rates, the memory requirements are rather modest. Already remembering a few interactions, either with the same co-player or across different co-players, may provide players with enough information to adopt reciprocal strategies.

### (d) Beyond reactive strategies and the donation game

While the results presented in the main text focus on reactive strategies and the donation game, the observed patterns hold more generally. To illustrate this point in more detail in the electronic supplementary material, we first consider the dynamics of the donation game among memory-1 strategies. Here, players take into account both their co-player’s and their own last move; see [66,67]. Also in that case, we observe that perfect memory leads to systematically higher cooperation rates (electronic supplementary material, figures S5 and S6). Again, this advantage of perfect memory is particularly pronounced for strong selection, or when there is a high benefit of cooperation (electronic supplementary material, figure S7).

In a second step, we also analyse the evolutionary dynamics of a different social dilemma, the snowdrift game [68]. In this game, individuals can again cooperate or defect. However, now they get the benefit *b* if at least one of them cooperates. Cooperators in turn pay a cost of *c* or *c*/2, depending on whether they are the only one to cooperate or not. Compared with the donation game, the snowdrift game represents a weaker form of a social dilemma, because defection is no longer the dominant action in any single round [69]. Again, we derive analytical results in the case of large populations and strong selection. For perfect memory, cooperation is stochastically stable when *q* < 1 − *c*/(2*δb*). Interestingly, for limited payoff memory, we obtain the same condition as for the donation game, *q* < 1 − 1/(2*δ*). We explain this invariance in detail in the electronic supplementary material: compared with the donation game, the ordering of one-shot payoffs in the snowdrift game only differs in one instance (the *sucker’s payoff* now exceeds the *punishment payoff*); however, this instance is irrelevant for the stochastic stability of cooperation. Further evolutionary simulations suggest that, again, perfect memory leads to higher cooperation rates than limited memory; but now even limited memory can result in substantial cooperation (electronic supplementary material, figures S8 and S9).

## 4. Discussion

In economics, if payoff is measured in terms of money and a decision is to be made at time *t* in the future, then currency accumulated early on is weighted more in that decision because it has more time to accumulate interest. Such a model discounts the future relative to the past. It also does not necessarily require ‘memory’ because rewards are accumulated into a factor used in decision-making; the specific time stamps of rewards do not themselves provide better information beyond their effects on total payoff. In a similar fashion, the probabilistic interpretation of discounting as a continuation probability [15] also effectively discounts the future relative to the past. A foraging animal deciding between two behaviours might tend to choose the one that yields a moderate reward sooner relative to a larger reward later, since earlier rewards (e.g. food) contribute to immediate survival, and there is no guarantee that later rewards will happen at all [70].

Within the context of a single repeated game, the model we consider here is, in some ways, dual to the classical model of temporally discounted rewards in repeated games. Instead of making decisions based on expected rewards in the future, we consider individuals who make decisions based on actual rewards in the past. Thus, the ability to estimate the future payoff of a strategy is replaced by the memory of how this strategy previously fared against others. This involves two time scales: interaction partners and rounds within those interactions. As a result, we are dealing with a model that discounts the past rather than the future. Intriguingly, treating payoffs in this manner is reminiscent of the reward-smoothing technique of ‘eligibility traces’ in reinforcement learning [71], which uses past rewards (discounted appropriately) to shape present perceived payoff. There is a sound basis for this method in neuroscience, where rewards and (temporal-difference) learning are associated with dopaminergic neurons [72] and spike-timing-dependent plasticity [73]. This suggests a more biologically encoded interpretation of memory, which is equally applicable to models of direct reciprocity where rewards have a neurological basis.

Of course, the precise nature of ‘memory’ also depends on what payoffs in a game represent, which should be taken into account when applying game-theoretic models. For example, a payoff stream of monetary currency might truly accumulate and not require memory on the parts of agents. Even in the context of money, however, not all of what was obtained in the past is necessarily available at the time a decision is made, which brings memory into play. The serial position effect in human psychology shows that in an ordered list of items (e.g. words), humans tend to have difficulty remembering the entirety of sequences, demonstrating moderate recall for those items coming earlier (primacy effect), substantial recall for those coming later (recency effect), and lower recall for those in between [74]. It is therefore reasonable that when presented with a stream of payoffs, whether on the time scale of pairing for repeated interactions or in a stream of one-shot games, players might be able to effectively incorporate only the most recent payoffs.

In fact, even beyond specific psychological considerations, a curious interpretation of payoffs arises from the formula commonly used for expected payoffs in repeated games. If *δ* ∈ [0, 1) is the probability of continuing to another round in the game, then the expected payoff to an agent is $(1-\delta )\sum_{t=0}^{\infty }\delta^{t}u_{t}$, where *u*_*t*_ is the reward the agent receives in the stage game at time *t*. Here, additional stochasticity arises owing to uncertainty in the game length, and an agent might not be able to compute his or her expected payoff for use in decision-making. The probability that the game terminates after the interaction at time *T* is *δ*^*T*^(1 − *δ*), in which case $(1-\delta )\sum_{t=0}^{\infty }\delta^{t}u_{t}$ is exactly the expected payoff the agent receives at time *T*, i.e. *in the last round*. As an unbiased estimator of this expectation, the agent might thus use *u*_*T*_ as a proxy for ‘success’ when evaluating his or her behaviour. This gives a purely model-driven justification for why considering payoff in the last round of the game can result in more realistic extensions of traditional models.

We note that the expected *total* payoff in the game, *u*_0_ + *u*_1_ + ... + *u*_*T*_, is given by $\sum_{t=0}^{\infty }\delta^{t}u_{t}$. This version of ‘expected payoff’ appears less common in the literature on direct reciprocity than its normalization, $(1-\delta )\sum_{t=0}^{\infty }\delta^{t}u_{t}$, likely owing to the fact that payoffs can grow arbitrarily large with sufficiently long time horizons (*δ* → 1^−^). Non-normalized payoffs interfere with selection intensity (*β*) in models of social imitation, which is (presumably) why they appear less frequently in the literature. On that point, we note that differences between realized and expected payoffs disappear in the limit of weak selection, which is known in a general setting [65]. Non-weak selection can introduce substantial differences between models with realized and expected payoffs [75], which is especially important to understand in models of social systems with cultural transmission [76].

Our main contribution is an application of these ideas to direct reciprocity, which is one of the key mechanisms to explain why unrelated individuals might cooperate [7]. According to this mechanism, cooperation pays if it makes the interaction partner more cooperative in future. To describe which strategies are most effective, the previous theoretical literature assumes that the evolutionary dynamics are driven by the players’ expected payoffs [29–44]. To the extent that strategies are learned (not inherited), this assumption seems to impose rather stringent requirements on the individuals’ cognitive abilities. In the most extreme case, individuals would have to remember (or compute) their payoffs against all population members, for all possible ways in which their repeated games may unfold. This assumption introduces a curious inconsistency in how these models represent an individual’s cognitive abilities. For playing their games, individuals are often assumed to only recall the outcome of the very last round. Yet to update their strategies, individuals are implicitly assumed to have a record of the outcome of all rounds, across all interaction partners.

It is natural to ask, then, to what extent perfect payoff memory is in fact required for the evolution of reciprocity. To this end, we consider a model in which individuals only remember the payoff of their very last interaction, or the payoffs of the last few interactions. By only considering an individual’s most recent experiences, the evolutionary process is subject to additional stochasticity. Strategies that perform well on average (across an entire repeated game and across many interaction partners) may still get replaced if the respective player happened to yield an inferior payoff in the very last round. A similar element of stochasticity has been previously explored in the context of one-shot (non-repeated) games [77–81]. This literature studies which strategies are selected for when individuals only interact with a finite sample of population members. In the respective models, individuals can only choose between two strategies. They can either cooperate or defect, and stochastic sampling affects which of these two strategies is favoured. Instead, in repeated games, players have access to a large set of strategies (in our case, all reactive strategies; [82]). Here, stochastic sampling does not only affect whether cooperative or non-cooperative strategies are favoured; it also affects *which* conditionally cooperative strategies are favoured.

To explore the effect of payoff memory, we only considered the simplest model of reciprocity. This leaves several possible directions for future work. For example, for both our analytical analysis and our simulations, we assumed that populations are *well-mixed*. As a result, any population member is equally likely to be a given player’s last co-player. A similar analysis for structured populations would be desirable, as population structure can often promote cooperation without an explicit need for reciprocity [48,83]. Similarly, we assumed that after identifying a suitable role model, players can easily imitate that role model’s strategy. Especially in repeated games, this assumption is less innocuous than it may appear. After all, a player can typically only observe the co-players’ actions, but not their underlying strategies. This player may only attempt to infer the co-players’ strategies from their past reactions. Once players only have a restricted recollection of the past, this task seems particularly difficult. In the context of our study, this might be a particular simplification, because players with limited payoff memory may also have less accurate information to infer a co-player’s strategy. Finally, we have taken a player’s payoff memory to be a given parameter of the model. Instead, future models could explore how that payoff memory might coevolve along with the players’ strategies (similar to previous work on the evolution of the players’ feasible strategy sets e.g. [43,49,52]). Here, individuals might experience an interesting trade-off. Better payoff memory would help them to make better decisions, yet it might also come with higher cognitive costs. For such a setup, our results suggest that in many instances, a modest amount of memory may be sufficient to achieve good outcomes.

## 5. Conclusion

Herein, we combine analytical methods and computer simulations to explore the impact of payoff memory on the evolution of reciprocal altruism. In the most extreme case, we consider individuals who update their strategies based on only one piece of information: the last round of a single repeated game. Compared with exisiting models where decisions are made based on expected payoffs (perfect payoff memory), we find that individuals are less generous, and they tend to be less cooperative overall (figure 1). However, once individuals update their strategies based on two or more recent experiences, overall cooperation rates quickly approach the levels observed under perfect payoff memory (figure 3). These findings suggest that models based on expected payoffs can serve as a useful approximation to more realistic models with limited payoff memory. Our findings also contribute to a wider literature that explores which kinds of cognitive capacities are required for reciprocal altruism to be feasible (e.g. [84,85]). While more payoff memory is always favourable, reciprocal cooperation can already be sustained if individuals have a record of two or three past outcomes. We believe that this kind of result, derived entirely within a theoretical model, is crucial for making model-informed deductions about reciprocity in natural systems.

## Data Availability

All data generated for this manuscript are openly available at: https://zenodo.org/records/10844104 [86]. The code for producing the results and generating the figures has also been archived and is available at: https://zenodo.org/records/11143305 [87]. Supplementary material is available online [88].
